# From STEMI to occlusion MI: paradigm shift and ED quality improvement

**DOI:** 10.1007/s43678-021-00255-z

**Published:** 2021-12-30

**Authors:** Jesse T. T. McLaren, H. Pendell Meyers, Stephen W. Smith, Lucas B. Chartier

**Affiliations:** 1 Department of Family and Community Medicine, University of Toronto, Toronto, ON Canada; 2 Emergency Department, University Health Network, Toronto, ON Canada; 3 Department of Emergency Medicine, Carolinas Medical Center, Charlotte, NC USA; 4 Department of Emergency Medicine, Hennepin County Medical Centre and University of Minnesota, Minneapolis, MN USA; 5 Division of Emergency Medicine, Department of Medicine, University of Toronto, Toronto, ON Canada; 6 Toronto General Hospital, 200 Elizabeth Street, R. Fraser Elliott Building, Ground Floor, Room 480, Toronto, ON M5G 2C4 Canada

**Keywords:** ST Elevation Myocardial Infarction, Electrocardiography, Quality Improvement, Emergency Department

A generation ago the ST-elevation myocardial infarction (STEMI) paradigm led to quality improvement (QI) in the emergency department (ED). Now, insights from angiography and advances in electrocardiogram (ECG) interpretation have led to the new paradigm of occlusion myocardial infarction (OMI), creating the possibility of further QI. This article reviews the current STEMI paradigm, the emergence of the OMI paradigm, and the use of QI to continuously improve care for acute myocardial infarction (AMI) patients in the ED.

## STEMI paradigm and QI

Thrombolytic therapy in the 1990s led to a paradigm shift in the treatment of AMI through emergent reperfusion. This changed the use of the ECG, from retrospectively classifying AMI into Q-wave/non-Q wave to prospectively identifying those with ST elevation, as a marker of AMIs with persistent occlusion without collateral circulation, which need emergent reperfusion. ED providers responded with QI initiatives to reduce reperfusion delays for AMIs with ST elevation, or STEMI, from emergency nurse-initiated ECG acquisition to emergency physician-initiated cath lab activation.

However, from the beginning of the STEMI paradigm there were questions about ECG interpretation at the heart of the diagnostic process. A 1994 report on ED delays published in *Annals of Emergency Medicine* summarized, “ECG abnormalities may be subtle or open to different interpretation, such as early repolarization or pericarditis. Only borderline or minimal ST-segment elevation may be present, and the emergency physician may be uncertain of its significance. The presence of left bundle branch block or left ventricular hypertrophy may complicate ECG diagnosis. The emergency physician may suspect that the ST elevation is old, but a previous ECG may be unavailable for comparison. The computer interpretation of the ECG on which some physicians rely may be incorrect. The emergency physician may not be sufficiently trained to recognize certain ECG patterns as signs of AMI” [1].

At the time little could be done to improve on these quality issues. Those that did not meet STEMI criteria were labeled “non-STEMI” (NSTEMI) and did not receive emergent reperfusion. But in the nearly 30 years since this paradigm emerged, insights from angiography and advances in ECG interpretation have identified the limits of this paradigm and given rise to a new one.

## From STEMI to OMI

Whereas the original thrombolytic trials were limited by rudimentary ECG analysis and AMI diagnosed by CK-MB (not angiography, and not even troponin), studies using angiography and formal STEMI criteria have put the paradigm to the test. For patients with STEMI, as adjudicated retrospectively by cardiologists, a recent prospective validation of STEMI criteria found that automated interpretation of the first ED ECG was only 35% sensitive for STEMI and 21% sensitive for any occlusion [2]. In a meta-analysis of 40,777 NSTEMIs in highly-monitored randomized-controlled trials, Khan et al. found a quarter of patients had a completely occluded coronary artery at the time of delayed angiography and had a nearly double mortality rate compared to NSTEMI patients with an open artery [3].

In response to these limitations, advances in ECG interpretation have identified signs of acute coronary occlusion that do not meet STEMI criteria. Emergency physicians such as Dr. Stephen Smith have played a leading role in these advances, which are summarized in his article in the *Canadian Journal of Cardiology* by Miranda et al. [4], and most recently in an article that provides step by step instructions in the diagnosis of OMI, and exclusion of mimics [5]. Examples include reciprocal ST depression in aVL, which can identify subtle inferior OMI and exclude pericarditis; a decision rule can differentiate between subtle left anterior descending (LAD) coronary artery occlusion and normal variant ST elevation in leads V2–V4; the modified Sgarbossa criteria can identify acute coronary occlusion in the presence of left bundle branch block and ventricular paced rhythms; the T/QRS ratio can differentiate LV aneurysm morphology from acute infarct; and primary ST depression maximal in V1-4 can identify posterior OMI. Table 1 demonstrates examples of these OMI ECG findings, and the full range can be found in these references by Miranda et al. [4] and Aslanger et al. [5].

**Table 1:**
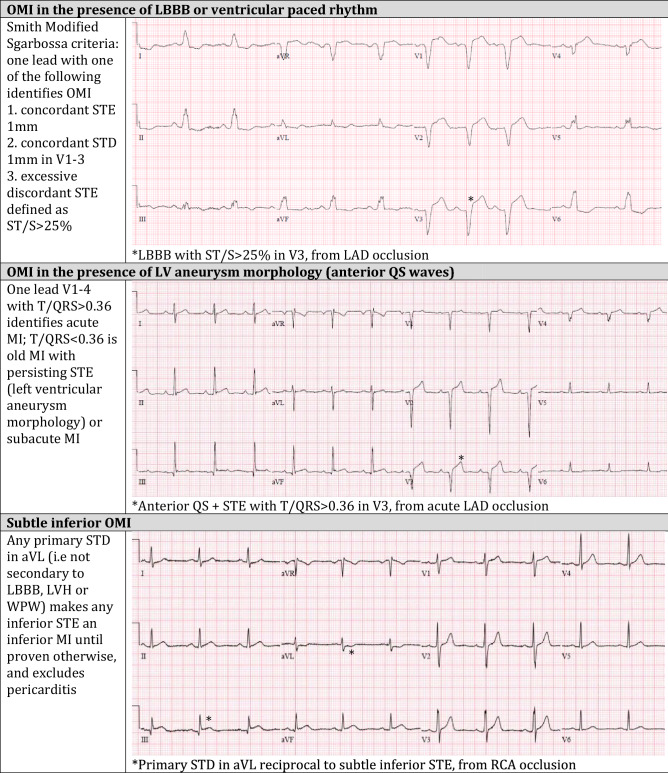

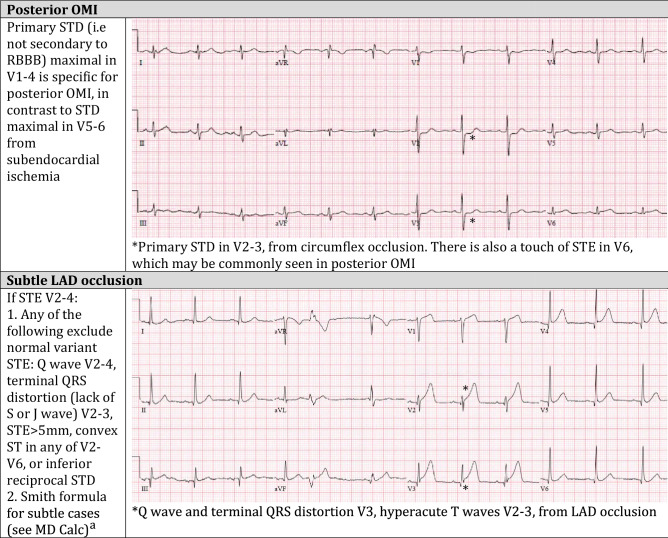
Examples of evidence-based criteria to identify occlusion myocardial infarction

References for evidence-based criteria can be found in the articles by Miranda et al. [4] and Aslanger et al. [5]

*OMI *occlusion myocardial infarction, *LBBB* left bundle branch block, *STE *ST elevation, *LAD* left anterior descending artery, *STD *ST depression, *LVH* left ventricular hypertrophy,* WPW *Wolf-Parkinston-White, *RCA* right coronary artery, *RBBB *right bundle branch block

^a^https://www.mdcalc.com/subtle-anterior-stemi-calculator-4-variable

These advances have given rise to a new paradigm, shifting the focus from the surrogate marker of ST segment millimeter criteria to the underlying pathology: occlusion MI [6]. Recent studies have now directly compared these paradigms. In the DIFOCCULT study, Aslanger et al. found that advanced ECG interpretation by cardiologists could reclassify 28% of NSTEMI as OMI, and this subgroup had a higher mortality rate than NSTEMIs whose ECGs had no evidence of OMI [7]. Meyers et al. showed that STEMI (+) OMI and STEMI (−) OMI have the same infarct size, mortality, number of wall motion abnormalities, and coronary interventions, which are significantly different than NSTEMI and especially NSTEMI that are non-OMI [8]. Furthermore, they found that emergency physicians expertly trained in ECG interpretation could identify OMI with twice the sensitivity as STEMI criteria, and significantly earlier [9].

These developments have answered the questions raised by *Annals* in 1994: computer interpretation and the STEMI paradigm on which it is based have limited accuracy for identifying acute coronary occlusion, evidence-based advances in ECG interpretation can differentiate between different causes of ST elevation and identify OMIs that do not meet STEMI criteria, and emergency physicians can be trained in this new paradigm. This new knowledge needs to be translated to the ED through QI approaches.

## OMI paradigm and QI

Among QI interventions, standardization and automation are higher on the hierarchy of effectiveness [10]. But we are currently operating with a paradigm based on a suboptimal standard, reinforced by inaccurate automation. All research, guidelines, and QI initiatives are designed only to improve care for patients with OMI that meet STEMI criteria on their ECG, ignoring those who don’t. Ultimately, we need to complete the paradigm shift, with OMI as the new standard, aided by artificial intelligence ECG interpretation of the totality of the ECG, not only the ST segments. Until that time, other QI interventions assume greater importance.

EDs should assess ECG and OMI quality benchmarks. A Door-to-ECG time of less than 10 min has been a key quality benchmark that has helped emergency nurses improve the speed of triage ECG acquisition through multiple QI interventions [11]. But there is a surprising lack of complementary quality benchmark for emergency physicians, perhaps because of simplified STEMI criteria. ECG-to-Activation time reflects the diagnostic time of emergency physicians, is independent of cath lab capabilities, and can be compared across different settings; this metric can help identify preventable reperfusion delays and promote new advances in ECG interpretation [12]. In our QI project, including a grand rounds presentation based on the article by Miranda et al., followed by weekly ECG audit and feedback to all physicians on signs of OMI, ECG-to-Activation time was reduced by 20 min [13].

EDs should review the ECG-to-Activation time (whether this activates their own cath lab or activates transfer to another centre’s cath lab) for all their patients with OMI. This includes the 25% or more of NSTEMI patients with occluded arteries on angiogram and the third of true STEMI patients that have an open artery by the time of angiogram. In order to identify all patients with an occluded artery at ED presentation, the definition of OMI includes the following: (1) confirmed OMI (angiographic culprit lesion with TIMI 0–2 flow), and (2) presumed OMI with significant cardiac outcome, defined as: (a) angiographic acute but non-occlusive culprit lesion with highly elevated troponin (as defined in several studies, between 70 and 300 times the 99th percentile upper reference limit, depending on the assay), (b) highly elevated troponin and new regional wall motion abnormality on echocardiography, in those without angiography, or (c) STEMI(+) ECG with death before angiogram [7–9]. EDs can design QI interventions based on this outcome, and target the different components of the reperfusion decision (Table 2).

**Table 2:** Comparison of quality improvement for STEMI and OMI paradigms

	STEMI paradigm	OMI paradigm
Quality improvement		
Outcome measure	STEMI criteria on ECG, with culprit lesion on angiography	All OMI on angiography: occluded arteries or open culprit arteries with very high troponins
ED quality metric	Triage: Door-to-ECG time Diagnosis: ECG-to- Activation time	Triage: Door-to-ECG time Diagnosis: ECG-to-Activation time
QI interventions		
Automation	ECG computer interpretation of STEMI criteria only	Artificial intelligence interpretation of ECG signs of OMI
Protocols	Code STEMI for patients with ECGs meeting STEMI criteria STAT cardiology consults for equivocal STEMI	Cath lab activation for patients with OMI based on clinical, ECG ± POCUS assessment STAT cardiology consults for equivocal OMI
Alerts	STEMI alert for STEMI criteria on ECG	Patient alerts for refractory ischemia
Reminders/checklists	Reminders/checklists of STEMI criteria only	Reminders/checklists of clinical, ECG, and POCUS signs of OMI
Audit/feedback	Audit STEMI cases only, feedback about STEMI criteria only	Audit all OMI cases, feedback about clinical, ECG and POCUS signs of OMI
Education/training	Education/training for ECG STEMI criteria only	Education/training for clinical, ECG, and POCUS signs of OMI

*STEMI *ST-elevation myocardial infarction, *OMI *occlusion myocardial infarction,* ED *emergency department, *QI *quality improvement, *POCUS *point-of-care ultrasound

ECG interpretation is a core competency for emergency medicine trainees [14]. It should be updated to include advances in OMI, which have been led by emergency physicians and empower emergency providers to better interpret ECGs at the bedside. As with point-of-care ultrasound (POCUS) skills [15], advanced ECG interpretation requires workshops and training to incorporate interpretation into clinical decision-making, in addition to ED administrative support and quality assurance. Moreover, the OMI paradigm shift is not just about the ECG. The entire outcome is changing from a single element of the ECG (i.e., certain ST elevation voltage) to a patient-oriented one (i.e. occlusion or not), and QI needs to reflect that. While POCUS is not needed for obvious STEMI(+)OMI and can unnecessarily prolong ECG-to-Activation time, advanced POCUS training can help identify regional wall motion abnormalities that complement subtle STEMI(−)OMI ECGs. Patient alerts for refractory ischemia could help identify OMI patients who require cath lab activation even in the absence of ECG changes (as current guidelines recommend). Protocols and audits of STAT cardiology consultations can help with joint decision-making for challenging cases that incorporate clinical, ECG and POCUS findings. Collaboration between emergency and cardiology departments on tracking OMI quality metrics, and implementing and assessing OMI quality improvement projects, can help emergency physicians and cardiologists advance towards the paradigm shift together.

## Conclusion

A generation ago, EDs responded to the STEMI paradigm through QI interventions that expedited the ECG acquisition and cath lab activation of patients with acute coronary occlusion that met STEMI criteria. Now insights from angiography and advances in ECG interpretation have led to the new paradigm of OMI. This creates the foundation for a new generation of ED quality improvement for all patients with OMI—including new outcome measures, new quality metrics, and new interventions based on clinical, ECG and POCUS findings. By engaging with the emerging OMI paradigm through the lens of QI, emergency providers can develop local initiatives and promote new standards of care.
